# Recapitulation of the embryonic transcriptional program in holometabolous insect pupae

**DOI:** 10.1038/s41598-022-22188-y

**Published:** 2022-10-20

**Authors:** Alexandra M. Ozerova, Mikhail S. Gelfand

**Affiliations:** 1 Skolkovo Institute of Science and Technology, Moscow, Russia; 2 Institute for Information Transmission Problems (Kharkevich Institute), RAS, Moscow, Russia

**Keywords:** Functional clustering, Evolutionary developmental biology

## Abstract

Holometabolous insects are predominantly motionless during metamorphosis, when no active feeding is observed and the body is enclosed in a hardened cuticle. These physiological properties as well as undergoing processes resemble embryogenesis, since at the pupal stage organs and systems of the imago are formed. Therefore, recapitulation of the embryonic expression program during metamorphosis could be hypothesized. To assess this hypothesis at the transcriptome level, we have performed a comprehensive analysis of the developmental datasets available in the public domain. Indeed, for most datasets, the pupal gene expression resembles the embryonic rather than the larval pattern, interrupting gradual changes in the transcriptome. Moreover, changes in the transcriptome profile during the pupa-to-imago transition are positively correlated with those at the embryo-to-larvae transition, suggesting that similar expression programs are activated. Gene sets that change their expression level during the larval stage and revert it to the embryonic-like state during the metamorphosis are enriched with genes associated with metabolism and development.

## Introduction

Hemi- and holometabolous insects differ in the magnitude of physiological and morphological changes during the metamorphosis. In hemimetabolous insects, embryogenesis typically ends up with an adult-like larva that further develops to the imago through sequential molts causing gradual shifts, with the wings and genitalia appearing during the adult molt. In holometabolous insects, the adult body plan is established at the prepupal and pupal periods, and larval organs and systems are de-differentiated and reorganized during the complete metamorphosis. This is usually accompanied by a more or less radical change in the habitat and feeding strategy. Larvae and adults of the same species do not share food resources, allowing the separation of growth and reproduction in time and space^1^.

Metamorphosis is believed to originate approximately 400 million years ago in the early Devonian, when Pterygota emerged, the insect flight was invented^2^, and complete metamorphosis evolved to support the ability to fly. Sequential molts require the whole body, including the wings, to be covered with the cuticle. It makes wings too heavy and almost no extant winged insects undergo molting during the imago stage, an exception being the short-living subimago of the mayfly that undergoes a full molting cycle to become the imago^3^.

During larval development some cells with latent embryonic potential are arrested and the differentiation process continues after the pupation^4^. These cells, initially forming so-called imaginal primordia, replace larval cells to form adult organs. The imaginal cells contribute little to the functioning of the larval organism and preserve pluripotency, similar to stem cells^5^. For example, in *Papilio xuthus* (Lepidoptera), a sophisticated orchestra of transcription factors that regulate the expression patterns of opsins, manifest only after the pupation to build the compound eye^6^. On the other hand, some organs undergo dedifferentiation followed by redifferentiation to the adult state. For example, in *Drosophila* (Diptera), syncytial alary muscles de-differentiate to mononuclear myoblasts prior to formation of the adult tissue^7^.

Differentiation of stem cells into mature tissues could reuse molecular mechanisms that drive the embryonic development, since the gain of new features is based on the upgrade of the existing ones^8^. Therefore, it could be hypothesized that the pupal gene expression program should resemble the embryonic one due to both differentiation d*e novo* and redifferentiation. A study on the midge *Polypedilum vanderplanki* showed reversion of the transcriptional profile back to the embryonic stage during metamorphosis^9^. Here, we comprehensively analyze all insect developmental transcriptome datasets available in the public domain, with the aim to assess gene expression similarities between pupae and embryos.

## Methods

### Datasets

Developmental transcriptomic datasets with at least one sample originating from each of the four major stages (embryonic, larval, pupal, adult) in the holometabolous insect development were analyzed. The collection includes ten species from four orders (Diptera, Hymenoptera, Lepidoptera, Coleoptera). For *Drosophila melanogaster*, both RNA-seq and microarray datasets are available, and RNA-seq only for all other species, see Table 1 and Supplementary Tables S1–S9 for details. Specific timing of samples, source tissue and sex are shown in Supplementary Tables S1–S9 if provided in the original papers.

**Table 1:** Datasets.

Species	Number of samples	Layout*	Mapped reads per sample**	Assembly accession (RefSeq if available)	Source
*Drosophila melanogaster* (fly)	12	PE	4.9M	GCF_000001215.4	^11^
*Drosophila melanogaster* (fly)	75	PE	30M	GCF_000001215.4	^12^
*Drosophila melanogaster* (fly)	153	microarray dataset, three platforms			^13^
*Bactrocera dorsalis* (fly)	4	SR	0.1M	GCF_000789215.1	^14^
*Zeugodacus cucurbitae* (fly)	4	PE	38.4M	GCF_000806345.1	^15^
*Zeugodacus cucurbitae* (fly)	52	PE	18.3M	GCF_000806345.1	^16^
*Megalopta genalis* (bee)	30	PE	9.2M	GCF_011865705.1	^17^
*Ostrinia furnacalis* (moth)	4	SR	19.8M	GCF_004193835.1	^18^
*Plutella xylostella* (moth)	4	PE	16.9M	GCF_000330985.1	^19^
*Manduca sexta* (moth)	26	PE and SR	8.9M	GCF_000262585.1	^20^
*Tribolium castaneum* (beetle)	12	SR	1.8M	GCF_000002335.3	^21^
*Polypedilum vanderplanki* (midge)	5	PE	8.8M	Scaffold v0.9^22^	^9^

* RNAseq datasets have either paired-end (PE) or single-read (SR) layouts.

** The numbers of mapped reads are given in millions (M).

Mature females contain eggs; therefore, full-body transcriptomes have a strong signal from the eggs, yielding a high correlation of the female samples with the embryonic state. To avoid these confounding effects, the female samples were excluded from the analysis.

After the initial analysis, the *Pieris rapae* dataset^10^ was excluded due to insufficient data for the embryonic sample, the latter being an outlier with only 0.2M uniquely mapped reads, compared to about 13M reads for each other sample.

The *Tribolium castaneum* dataset comprises three replicates for each developmental stage. Two of the pupal samples had exactly the same set of raw reads; therefore, one copy was excluded.

### RNAseq preprocessing

RNA sequencing reads were downloaded from the NCBI sequence read archive in the sra format, and fastq files containing reads were extracted. Low-quality reads that had low average nucleotide quality, shorter length than expected or high number of missing nucleotides were eliminated using the *fastp* tool^23^. Automatically detected adaptors (based on read overlapping analysis and built-in known adapter sequences from the *fastp* package) and low-quality regions at the end of the reads were trimmed.

For each organism, a reference transcriptome index was prepared by the *Kallisto index* tool^24^, see accessions of the published transcriptomes in Table 1. For both single-end and paired-end reads, mapping was performed using a pseudo alignment approach implemented in the *Kallisto* package. The FPKM (Fragments Per Kilobase Million) normalization was used for the downstream analysis.

### Microarray dataset

Processed data containing log-transformed ratios between channels were downloaded from the NCBI GEO database (accession GSE3286) for three platforms separately. Probe IDs were converted into gene names using microarray specification information from NCBI GEO (related platforms are deposited under GPL2837, GPL2838 and GPL2840 accessions) and *D. melanogaster* gene information from the FlyBase database^25^.

### Gene Ontology (GO) terms annotation

The *D. melanogaster* GO annotation was downloaded from the AmiGO 2 database^26^ for all three aspects (Biological Process, Molecular Function and Cellular Component).

*InterProScan* with the default parameters was used to predict GO terms for other species^27^. Protein sequences from the respective assemblies (see Table 1) were used as an input for *InterProScan*.

Genes were assumed to be related to development, if the Gene Ontology (GO) term “developmental process” (GO:0032502) or its descendant terms were predicted to be associated with the gene. These genes comprised the development-associated gene subset that was used further in the analysis. Genes predicted to have the GO term “metabolic process” (GO:0008152) or its descendants were regarded as metabolism-related. These genes comprised the metabolism-associated gene subset.

### Across-stages similarity

Similarity between stages was measured by the Spearman correlation coefficient of the log-transformed FPKM values.

### Random sampling

To assess the influence of particular gene subsets on the observed transcriptome characteristics, random sampling was performed. At that, gene sets of the same size as the selected gene set were randomly sampled several times. For each random gene set the desired metric was calculated. The obtained distribution of the possible metric values was used as a reference distribution to estimate the p-value or quantile of the observed data.

### Gene profile clustering

For datasets with data available for four main stages only, there are 27 possible patterns of gene expression (it could increase, decrease or remain the same during each of three transitions between stages). A gene was assigned to the pattern with which it had the highest correlation. Thus, for such datasets, genes were divided into 27 clusters. We were specifically interested in two clusters corresponding to the zigzag pattern of gene expression across the development, where the transcriptome profile reverts back to the embryonic state during metamorphosis.

The transcriptome datasets with more than four time points were hierarchically clustered with the Spearman correlation coefficient as the distance metric. The hierarchy algorithm from the *scipy* package was used^28^.

### GO term enrichment analysis

Python package *goatools* was used to identify significantly enriched GO terms^29^ using the default parameters with an adjusted *p*-value threshold equal to 0.05. The set of all genes with positive estimated expression values in the corresponding dataset was used as the reference set for GO enrichment analysis.

### Visualization

Python 3.7, *matplotlib* and *seaborn* packages were used for the visualization. Plots for the semantic analysis of the GO enrichment results were generated using the *REVIGO* tool^30^ and the R *ggplot* package.

## Results and discussion

### Intra-species comparison

**Figure 1:**
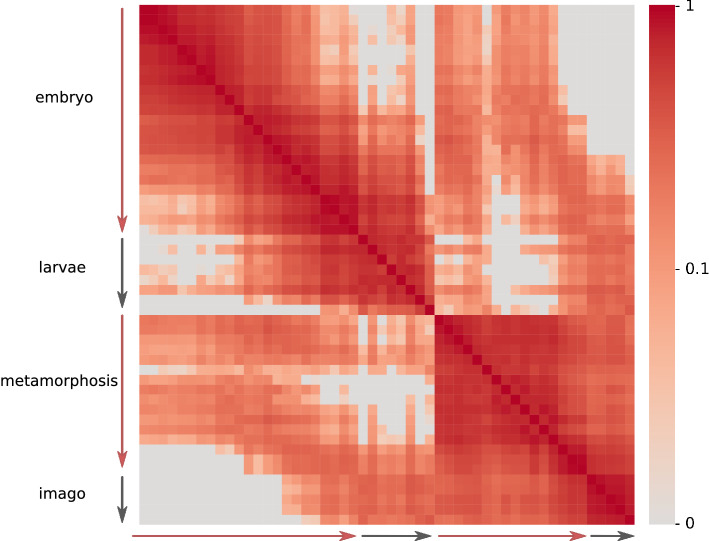
Pairwise correlation coefficient heatmap for *D. melanogaster* developmental stages. Sequential stages of development are shown on both axes with arrows indicating the four major stages. The color of each cell reflects the Spearman correlation coefficient of gene expression profiles for the respective developmental stages: the brighter is the cell, the higher is the correlation coefficient. The heatmap is symmetric with respect to the diagonal. The expression data are from^13^.

Intra-species comparisons across developmental stages allowed us to compare the transcriptome profiles at several distinct time points. A monotonic development results in gene expression patterns at each particular stage being closest to the immediately previous and following developmental stages, yielding a decrease of the similarity with the increase of the time interval between the time points. Indeed, this behavior is observed for the embryonic and larval stages. The correlation coefficient decreases for relatively more distant stages, as seen in the pairwise correlation heatmap for the detailed *D. melanogaster* dataset (Fig. 1). In that case, high Spearman correlation coefficients are concentrated near the diagonal.

However, this monotonic development is interrupted during the pupation suggesting drastic changes in the transcriptome profile. Gene expression levels at early prepupal and pupal stages are closer to the embryonic profiles rather than to the larval ones. It suggests some crucial event to happen during prepupal stages that will drive development towards formation of the adult body. An example of such an event could be the loss of the juvenile hormone that is thought to reactivate morphogenesis^31^. Moreover, in *Manduca sexta*, the level of the juvenile hormone decreases significantly before entering the prepupal stages (to trigger cell proliferation) with a narrow burst of the hormone titre during the prepupal development (to prevent precocious adult development)^32,33^.

The monotonic development is restarted at some point during the metamorphosis, extending to the adult stages, so that high correlation coefficients are again observed close to the diagonal of the matrix.

**Figure 2:**
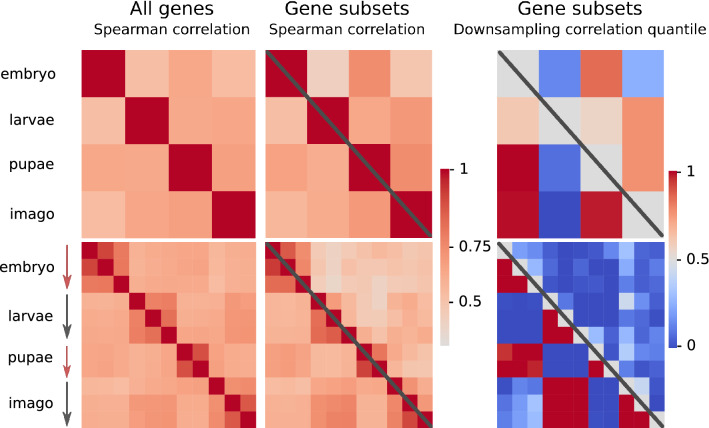
Pairwise correlation analysis for *O. furnacalis* (top) and *T. castaneum* (bottom). Sequential stages of development are depicted on both axes for each plot. The Spearman correlation coefficients were calculated for each pair of samples in each species: brighter cells correspond to higher correlation coefficients (left and middle). Symmetric matrices for the correlation coefficients for all genes are shown on the left. Correlation coefficients for the development-associated gene subset (the upper triangle, above the diagonal, for each species) and the metabolism-associated subset (the lower triangle, below the diagonal, for each species) are shown in the middle column. The results of random sampling analysis (see Methods) considering the development-associated gene subset (the upper triangle for each species) and the metabolism-associated gene subset (the lower triangle for each species) are given in the right column: high quantile values yields statistical support to the observed correlation being higher than expected for a random gene subset.

Datasets from several other insect species, though less detailed, demonstrate the same overall pattern, with pupal transcriptomes being more similar to the embryonic ones than to the larval or adult ones. Sample heatmaps for moth *Ostrinia furnacalis* and beetle *T. castaneum* are presented in Fig. 2 (left). Heatmaps for other datasets see in Supplementary Fig. S1.

**Figure 3:**
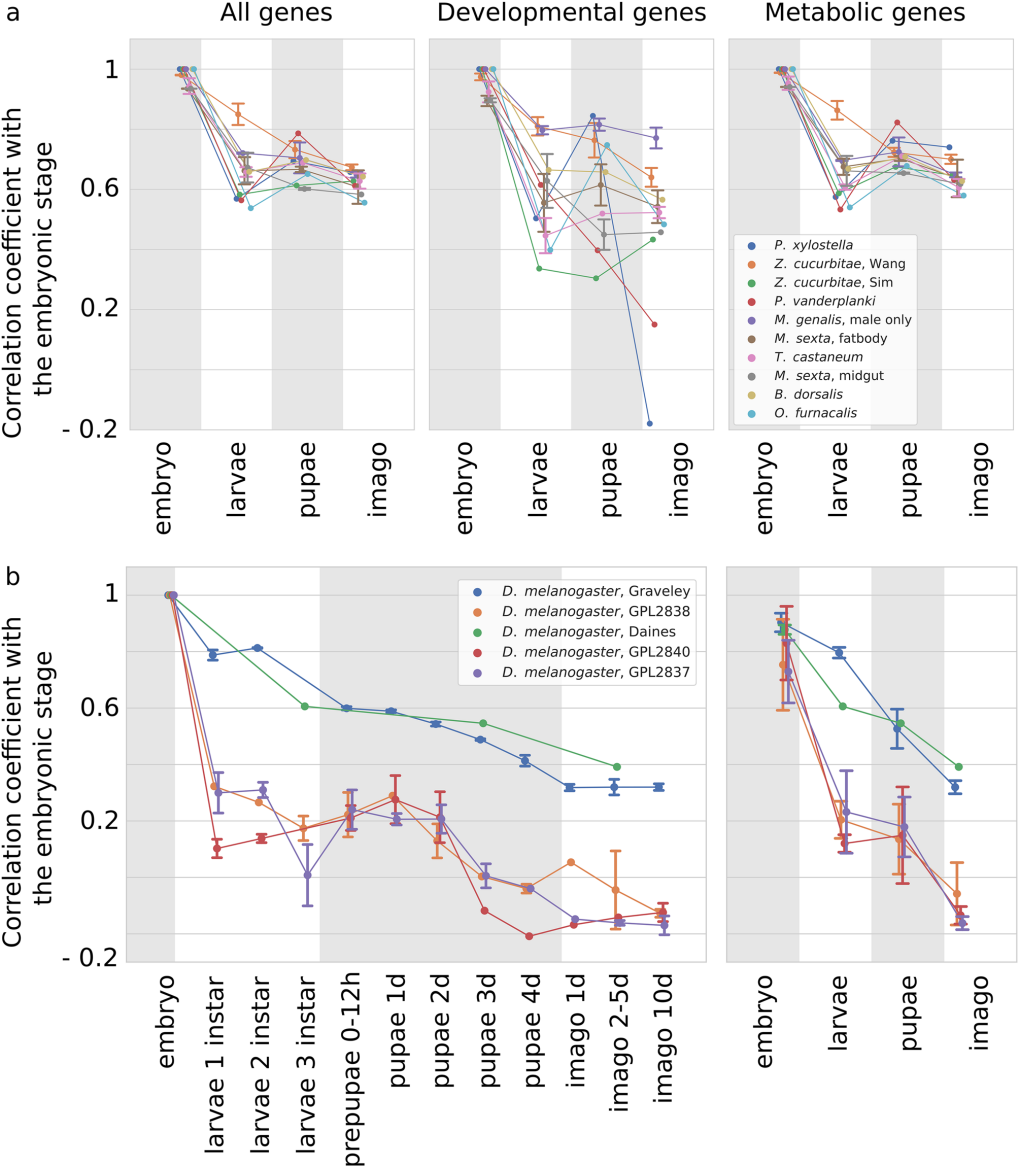
Similarity with the embryonic transcriptional profile. Sequential stages of development are shown on the horizontal axis, the correlation with the embryonic state is shown in the vertical axis. Quartiles are shown for datasets with available replicates. The color of the lines reflects the source dataset, see the legend insert. (**a**) Correlation coefficients for all genes (left), development-associated gene subset (middle) and metabolism-associated gene subset (right) for all species excluding *D. melanogaster*. (**b**) Correlation coefficients for all genes for *D. melanogaster* datasets for the detailed data (left) and averaged across the four major stages (right).

The effect of increased similarity between embryo and pupa compared to the embryo-larvae similarity is observed in several more datasets (Fig. 3a, left); therefore, it is not restricted to the *Drosophila* genus or the Diptera order. However, for some species pupae do not resemble embryos, an example being *M. sexta* (Fig. 3a, gray line). This could be explained by the fact that the *M. sexta* samples were collected from the whole body for early developmental stages and from several specific tissues for later stages. This makes direct transcriptome comparisons less reliable, since differences could be tissue-specific regardless of the developmental stage. In other cases, a possible explanation is that early or late pupae have been collected, closer to the adjacent larval or adult stages, respectively.

The latter explanation is supported by three *D. melanogaster* microarray datasets (Fig. 3b). Indeed, while middle pupal stages are more similar to the embryonic stages, late pupae are more similar to adults.

*D. melanogaster* datasets fall into two groups depending on the technology used to generate the source data. The RNA sequencing datasets (blue and green lines in Fig. 3b) demonstrate a monotonic decrease in similarity with the embryo in the course of development.

### Functional subsets of genes

To understand the molecular basis of the observed pattern, functional subsets of genes were considered. During metamorphosis, tissues are reorganized or even developed de novo from stem cells, like in embryogenesis. From the lifestyle point of view, the pupa also resembles the embryo since it is motionless and lacks active feeding. Therefore, genes with Gene Ontology (GO) terms related to “developmental process” (GO:0032502) and “metabolic process” (GO:0008152) were tested to account for the observed effect.

For *D. melanogaster* RNA-seq datasets, the analysis of subsets yields largely similar results. For two detailed microarray datasets, genes associated with metabolic processes demonstrate higher correlation between the embryonic and pupal samples. However, the range of the interval between quartiles makes the observation unreliable.

Similarity between embryo and pupa is higher, when calculated based on gene subsets, rather than the entire dataset for the *O. furnacalis* (Fig. 2, middle top). To test the statistical significance of the finding, random sampling was performed (see Methods). A high quantile of the observed correlation coefficient is expected for gene subsets strongly influencing the effect. On the contrary, gene subsets that have expression patterns following the average trend would have quantile values close to one-half. High correlation between embryonic and pupal transcriptomes in *O. furnacalis* data is supported by quantile values (Fig. 2, right top), suggesting that both development and metabolism-associated genes are collinearly expressed during these stages.

On the other hand, in *T. castaneum*, the expression of development-associated genes does not follow this trend (Fig. 2, middle bottom). At that, it should be noted that *D. melanogaster* is the only analyzed species with verified GO terms annotation from a dedicated database, while the GO annotation for other species is predicted using *InterProScan*. Moreover, only 1% of all proteins are annotated as associated with GO:0032502 (“*developmental process*”), leading to noisy results (Fig. 3a, middle). On the contrary, as many as 40% of proteins in each species are assigned with GO:0008152 (“*metabolic process*”) and hence the correlation coefficients naturally are close to those obtained using the complete datasets (Fig. 3a, right). However, for both types of subsets, there are species with an enhanced effect.

### The reversion of the transcriptome pattern

**Figure 4:**
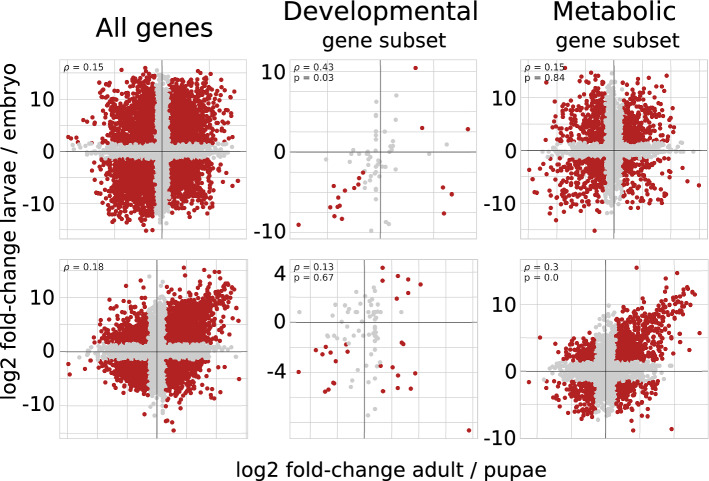
Changes in transcriptome profiles during the transition from the pupal to the imago stages compared to the transition from the embryonic to the larval stages for *O. furnacalis* (top) and *T. castaneum* (bottom). Each dot represents one gene, with the fold-difference between the embryo and larva expression in the y-axis and the fold-difference between the pupa and imago in the x-axis (log scales). Genes in the upper right corner of each plot have lower expression in embryo and pupa when compared to larva and imago, respectively. Three gene sets are considered: all genes (left), development-associated genes (middle) and metabolism-associated genes (right). Genes with significant LCF (greater than 1.5) are shown in red.

As described above, the transcriptome profile of holometabolous insects tends to revert to the embryonic state during metamorphosis. This can be seen not only from direct comparison of transcriptomes on several developmental stages, but from the analysis of transcriptome changes during transitions between adjacent stages. Indeed, in some cases, changes in gene expression that happen at the pupa-to-imago transition recapitulate the egg-to-larva transition. For example, the left part of Fig. 4 shows changes in gene expression for the *O. furnacalis* and *T. castaneum* datasets. A positive correlation between fold-change values is observed in 75% of the datasets. 40% of them have a correlation coefficient higher than 0.1, suggesting that the metamorphosis developmental program that drives (re-)formation of tissues and organs indeed dynamically recapitulates the embryo differentiation.

The effect of synchronized changes in expression patterns during embryo and pupal eclosion could be explained by monotonous processes occurring in the complete course of development. However, it could not be the main explanation, since in pairwise comparisons gradual changes are not observed and the pupal transcriptome is more similar to the embryonic rather than larval one.

**Figure 5:**
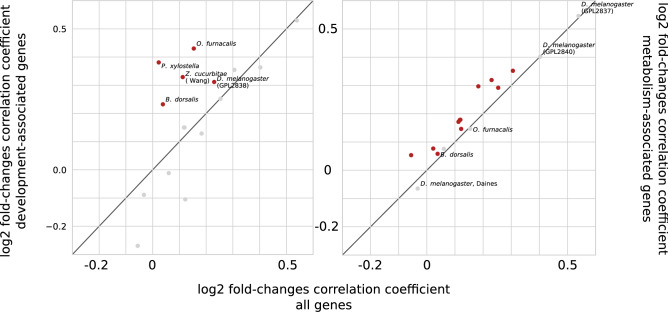
Correlation between transcriptome transitions from the embryo to the larva and from the pupa to the adult for all species. The correlations are calculated on the entire dataset (horizontal axis), the development-associated subset (according to gene ontology, vertical axis, left) and the metabolism-associated subset (vertical axis, right). Dots corresponding to the datasets with the statistically significant correlation coefficient are shown in red. Diagonal shows no changes in correlation coefficient when considering a gene subset instead of all genes.

For *O. furnacalis*, the development subset demonstrates higher correlation between fold-changes (middle top of the Fig. 4). Downsampling p-value supports this observation, since only 1% of random gene subsets show higher correlation. However, due to the low number of genes with predicted links to development, a significant effect is seen in only five datasets with positive correlation (Fig. 5, left).

Higher than average fold-change correlation for metabolism-associated genes is observed across more datasets (Fig. 5, right), and it is statistically significant for most of the species. Therefore, metabolic genes could partially drive the recapitulation.

Genes that drive recapitulation should have a zigzag-like pattern of gene expression during development. To identify such genes, clustering of the expression profiles was performed. Datasets with one time point measured for each of the main stages (embryo, larva, pupa and imago) are scored by a correlation coefficient with one of the possible 27 artificial trajectories (with up/same/down steps).

**Figure 6:**
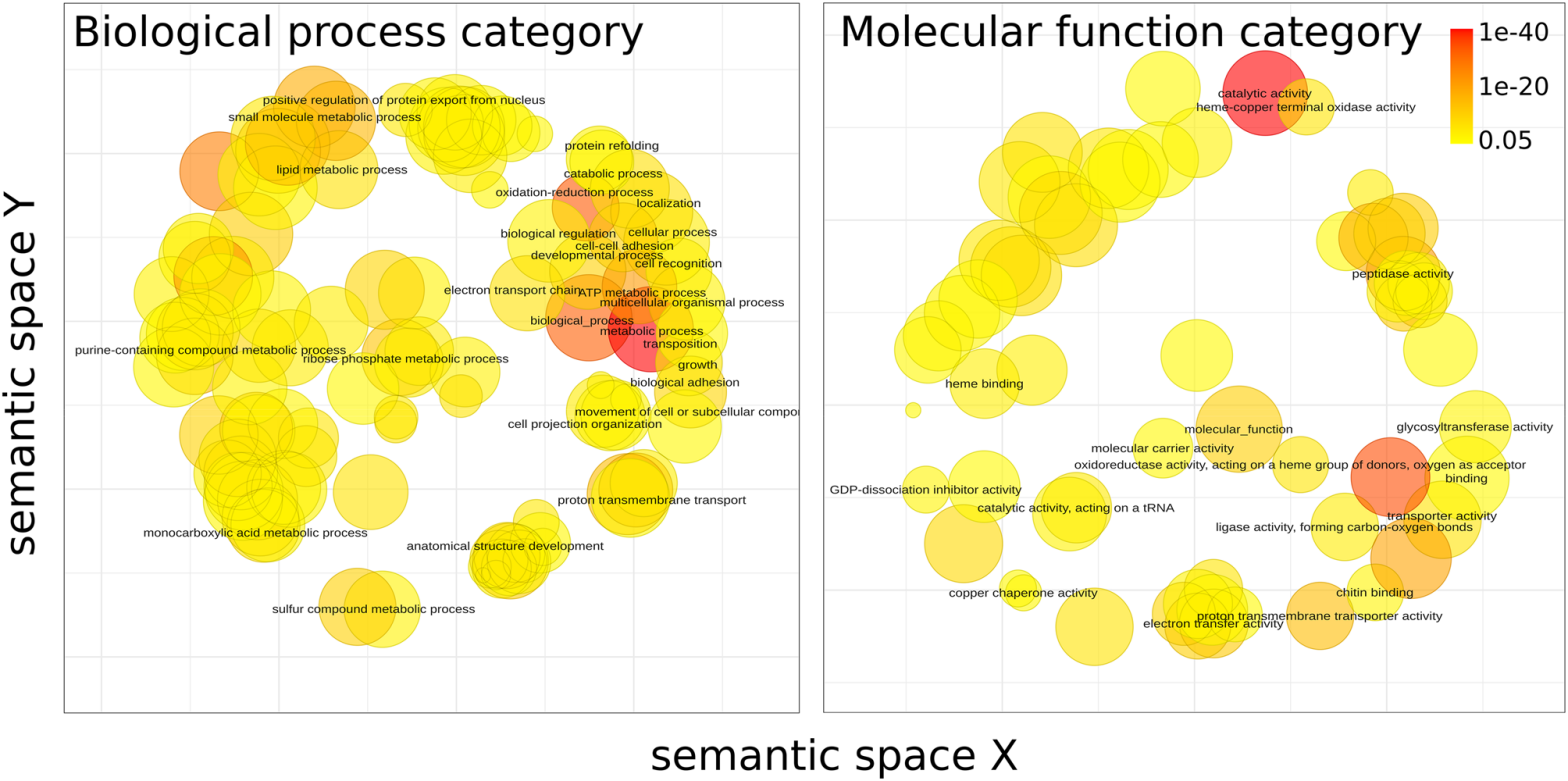
Gene ontology enrichment for genes primarily expressed during the embryonic and pupal stages. GO terms from the Biological Process aspect (left) and the Molecular Function aspect (right) are projected so that semantically close terms are spatially close. The color represents the adjusted p-value, multiplied over all the datasets. The size of circles reflects the log-transformed number of the term in the EBI GOA database^30^.

More detailed datasets were clustered hierarchically (see Methods). The *M. sexta* dataset was not considered at this step since 72% of its samples correspond to the larval stage and therefore the cluster diversity is dominated by gene expression changes during the larval development. For other datasets, clusters with the pattern of similar expression in the embryonic and pupal samples (zigzag-like pattern) were selected for further analysis (Supplementary Fig. S2). The set of genes with expression that increases while entering the larval stage and then decreases after the pupation is enriched with several classes of metabolism and development-associated terms (Fig. 6).

Terms similar to *purine containing compound metabolic process* (GO:0072521), *ribose phosphate metabolic process* (GO:0019693), *ATP metabolic process* (GO:0046034), *electron transport chain* (GO:0022900), *proton transmembrane transport* (GO:1902600) and o*xidation–reduction process* (GO:0055114) in the semantic space for biological processes category suggest a high rate of energy-generation and consumption during both embryogenesis^34^ and pupa maturation to build organs and tissues.

The enriched *peptidase activity* (GO:0008233) molecular function could be involved in several processes. For example, matrix metalloproteinases regulate trachea and intestinal development in embryo and pupal morphogenesis of *T. castaneum* beetle^35^; caspases, along with other proteases are key players in the induced cell death during metamorphosis, it is essential for remodeling of the larval tissue^36^. Peptidases also balance proliferation, being, in particular, crucial players in development of the tracheal system^37^ or neuroblasts^38^ in *D. melanogaster*.

**Figure 7:**
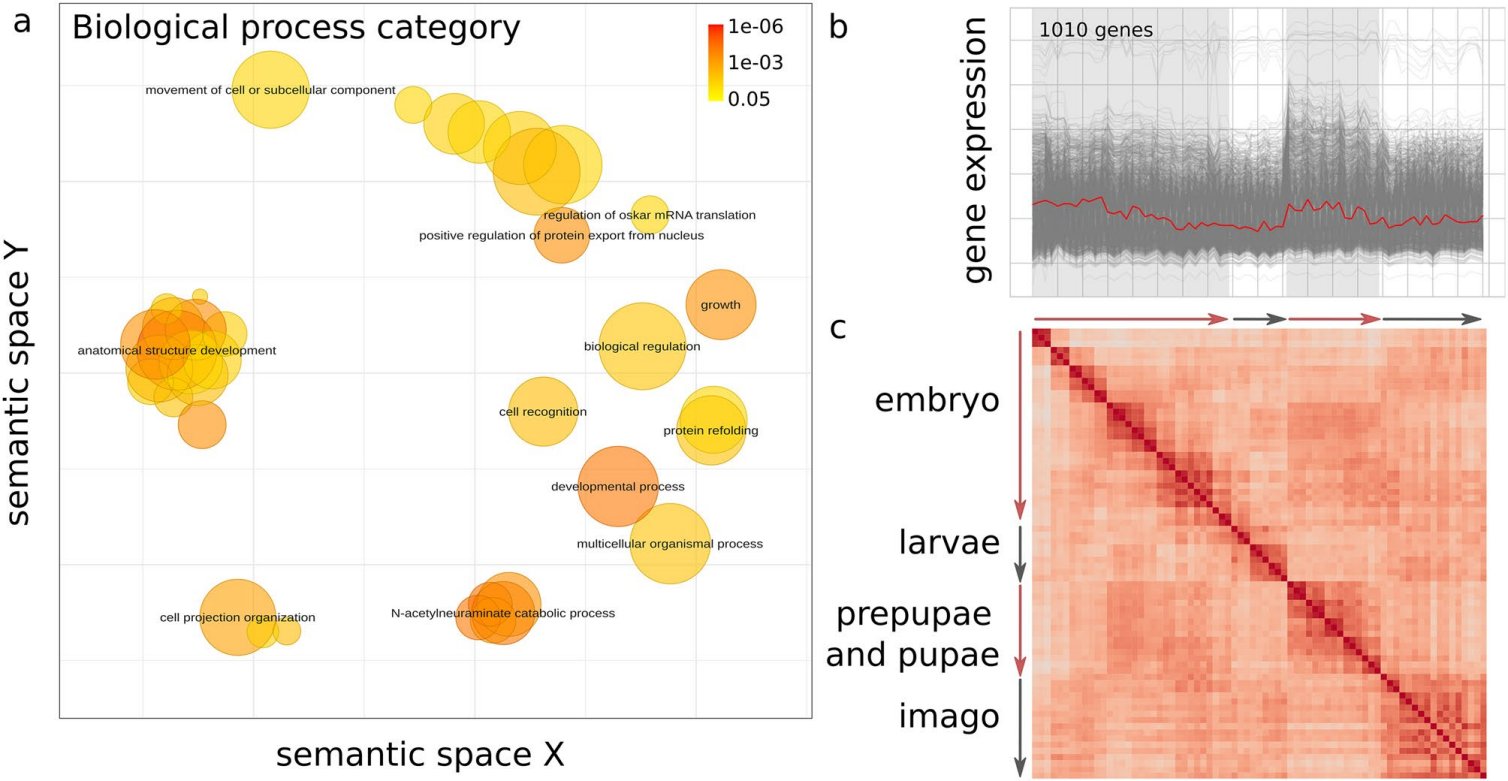
Analysis of the cluster with the zigzag pattern of gene expression in the *D. melanogaster* dataset. (**a**) GO enrichment results for genes comprising the cluster. GO terms from the Biological Process aspect are projected so that semantically close terms are spatially close. The color represents the adjusted p-value. The size of circles reflects the log-transformed number of the term in the EBI GOA database^30^. (**b**) Gene expression patterns across the development for the selected cluster. (**c**) Pairwise Spearman correlation coefficients for genes from the selected cluster.

*Cell–cell adhesion* (GO:0098609), *multicellular organismal process* (GO:0032501) and *anatomical structure development* (GO:0048856) terms are frequent among active genes during the embryo and pupal stages. In particular, these terms were enriched in the respective cluster in the *D. melanogaster* RNA-seq dataset^12^ (Fig. 7a and b, respectively). The correlation heatmap for this cluster features a distinct diagonal, suggesting each stage is similar to the ones that are close in time (Fig. 7c). However, there is a prominent diagonal in the embryo-pupae submatrix suggesting involvement of similar processes.

## Conclusions

Several datasets indeed show an increased similarity between the embryonic and pupal stages on the gene expression level when compared with the embryo-larvae transcriptome pairs. Sets of genes changing their expression level during the larval stage and returning to the embryonic state during the metamorphosis are enriched with genes related to energy metabolism and multicellular organism development.

Gene expression changes at transition from the embryonic to the larval stage for some datasets are correlated with changes between pupa and imago, suggesting similarity of transcriptional programs during embryonic development and pupal maturation. Separate analysis of metabolism-associated genes and genes related to the development enhances the observed effect for most datasets.

However, some datasets do not follow the pattern of embryonic expression recapitulation during morphogenesis. This might be due to the timing of collected pupal stages, as early pupae naturally resemble late larvae, while late pupae are similar to imagoes. Still, we consider the hypothesis to be tentatively confirmed and submit it for detailed experimental validation.

Two main opinions regarding the origin of metamorphosis origin in evolution are discussed in the literature. The Hinton hypothesis proposes the pupa to arise from the final nymphal instar of a hemimetabolous ancestor^39^. An alternative hypothesis suggests the larva of holometabolous insects represent an arrest phase of embryonic development, therefore metamorphosis is a continuation of embryogenesis^40^, a suggestion traced back to William Harvey^41^. In that case the pupa would correspond to all nymphal instars of hemimetabolous insects. The latter hypothesis is not supported by our observations, since it implies gradual development with a stalled larval stage without drastic changes during the prepupal and pupal period, contrary to our findings. However, although it is clearly a derived trait, as in most other Diptera the pupa is motionless, it might mean that some parts of the larval regulatory network are still active. At that, one might even expect that a detailed transcriptome analysis with good temporal resolution in a sufficient number of diverse species would demonstrate that both explanations are true to some extent, with the balance between continuous, monotonic developmental program (implied by the Harvey hypothesis) and considerable change in the transcriptome (as in the Hinton hypothesis) with partial recapitulation of the early embryonic transcription program (as observed here) would shift in different species and for different functional subsystems.

## Supplementary Information

Supplementary Information 1.

Supplementary Information 2.

Supplementary Information 3.

## Data Availability

The datasets analyzed in this study are available in the NCBI Gene Expression Omnibus under the accession GSE3286 — *D. melanogaster* microarray dataset^13^ and in the Sequence Read Archive under accessions SRA009364^12^, SRA012173^11^, SRP053022^14^, SRP045846^15^, SRP220120^16^, SRP057750^17^, SRP059012^10^, SRP070854^18^, SRP006371^19^, SRP047236^20^, SRP065255^21^ and DRP002405^9^ — RNA sequencing datasets.
