# Supplementary material for: Recapitulation of the embryonic transcriptional program in holometabolous insect pupae
Source: Sci Rep. 2022 Oct 20;12:17570. doi: 10.1038/s41598-022-22188-y (PMC9584902; doi:10.1038/s41598-022-22188-y)

Supplementary Figure S1. Pairwise correlation analysis.

Sequential stages of development are depicted on both axes for each plot. The Spearman correlation coefficients were calculated for each pair of samples in each species: brighter cells correspond to higher correlation coefficients (left and middle). Symmetric matrices for the correlation coefficients for all genes are shown on the left. Correlation coefficients for the development-associated gene subset (the upper triangle, above the diagonal, for each species) and the metabolism-associated subset (the lower triangle, below the diagonal, for each species) are shown in the middle column. The results of random sampling analysis (see Methods) considering the development-associated gene subset (the upper triangle for each species) and the metabolism-associated gene subset (the lower triangle for each species) are given in the right column: high quantile values yields statistical support to the observed correlation being higher than expected for a random gene subset.

D. melanogaster, Daines

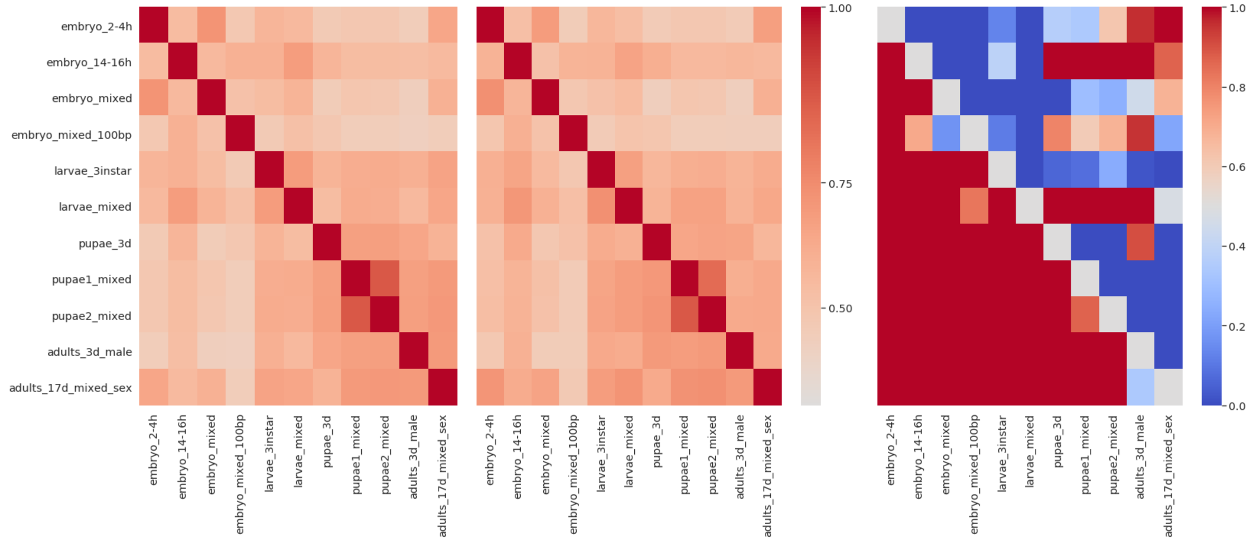

D. melanogaster, Graveley

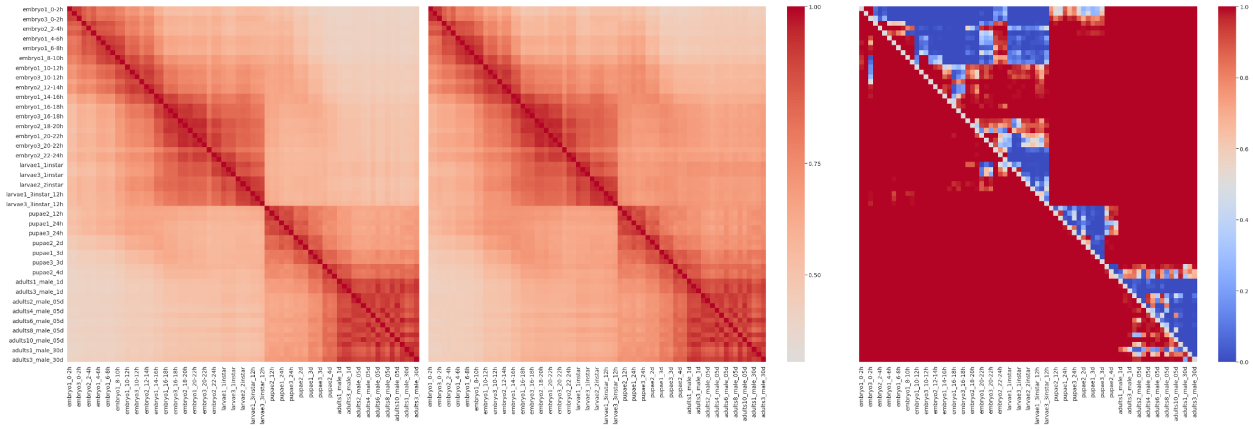

D. melanogaster, Arbeitman, GPL2837

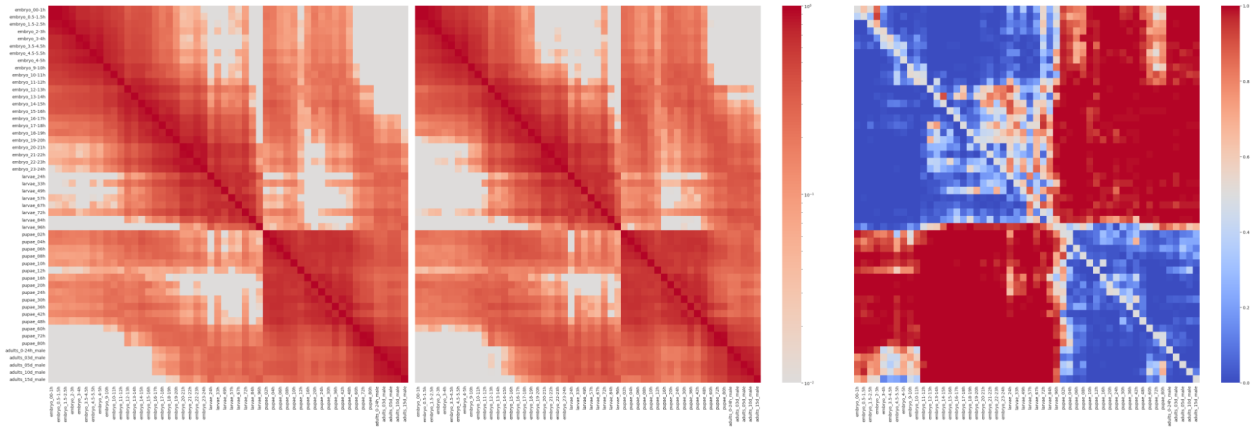

D. melanogaster, Arbeitman, GPL2838

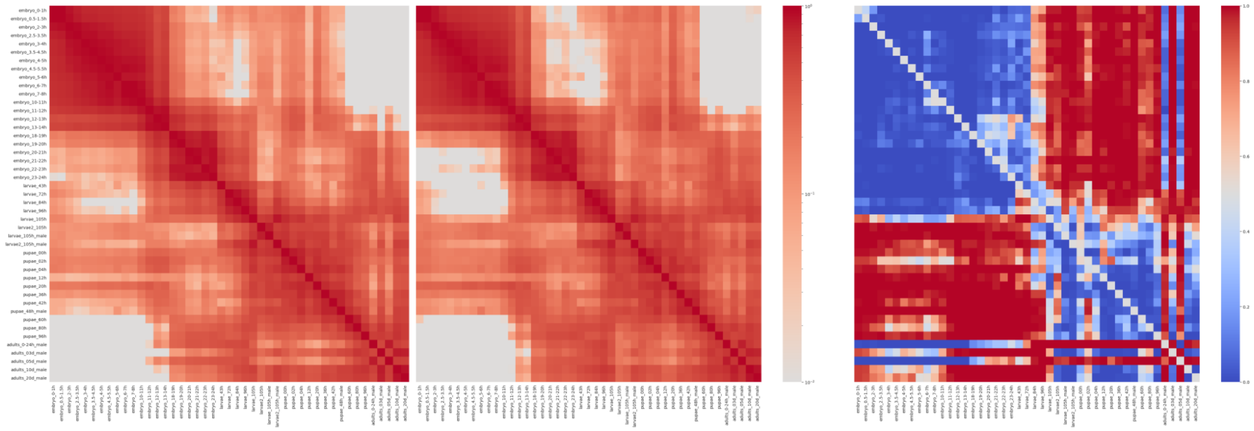

D. melanogaster, Arbeitman, GPL2840

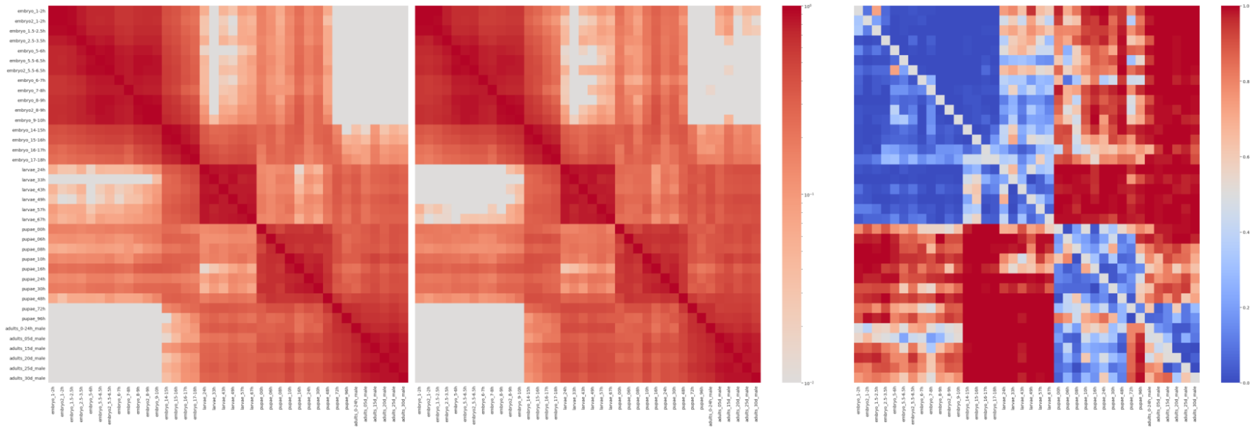

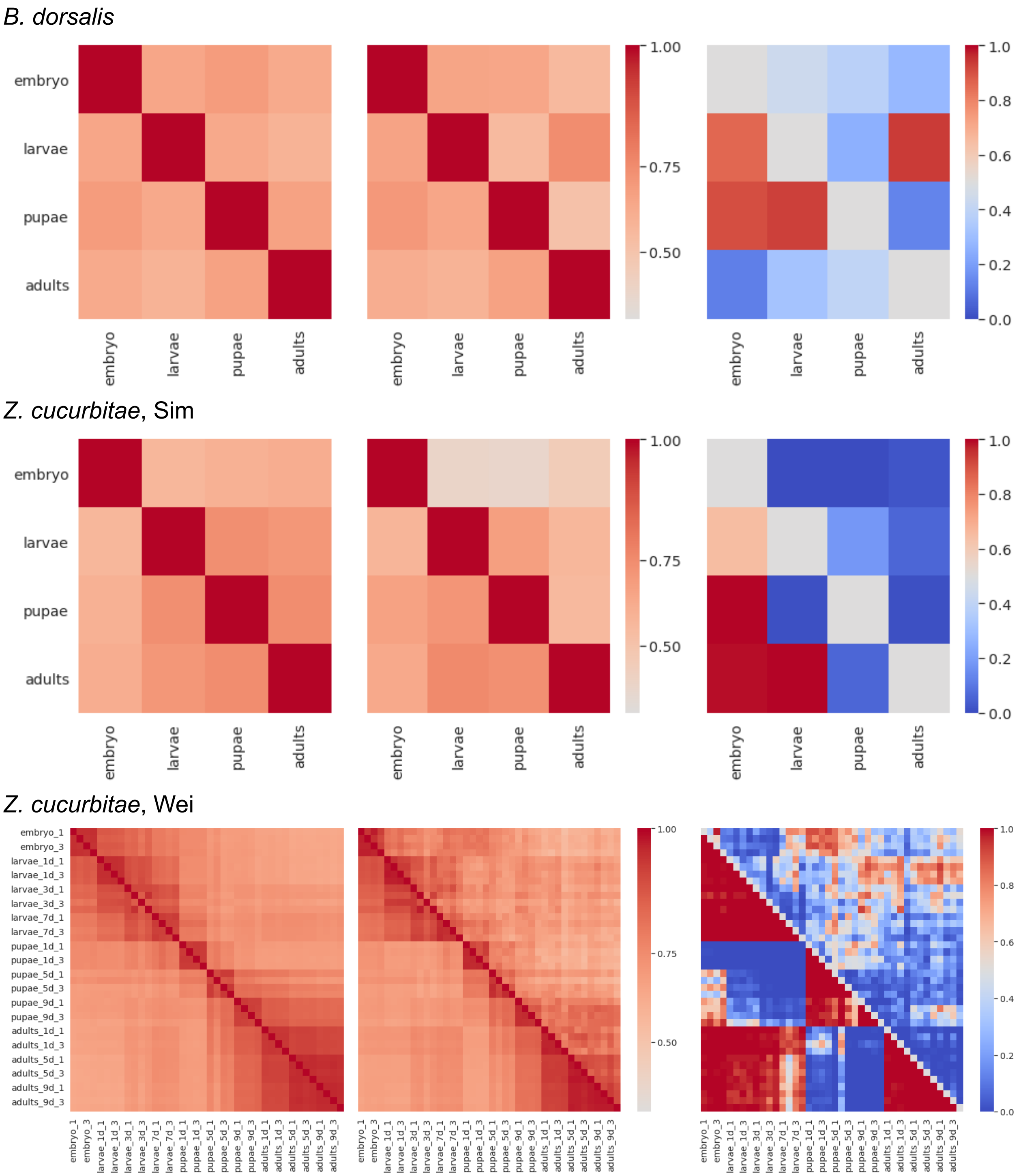

B. dorsalis
embryo
larvae
pupae
adults
1.00
0.75
0.50
1.0
0.8
0.6
0.4
0.2
0.0
Z. cucurbitae, Sim
Z. cucurbitae, Wei
embryo_1
embryo_3
larvae_1d_1
larvae_1d_3
larvae_3d_1
larvae_3d_3
larvae_7d_1
larvae_7d_3
pupae_1d_1
pupae_1d_3
pupae_5d_1
pupae_5d_3
pupae_9d_1
pupae_9d_3
adults_1d_1
adults_1d_3
adults_5d_1
adults_5d_3
adults_9d_1
adults_9d_3

M. genalis

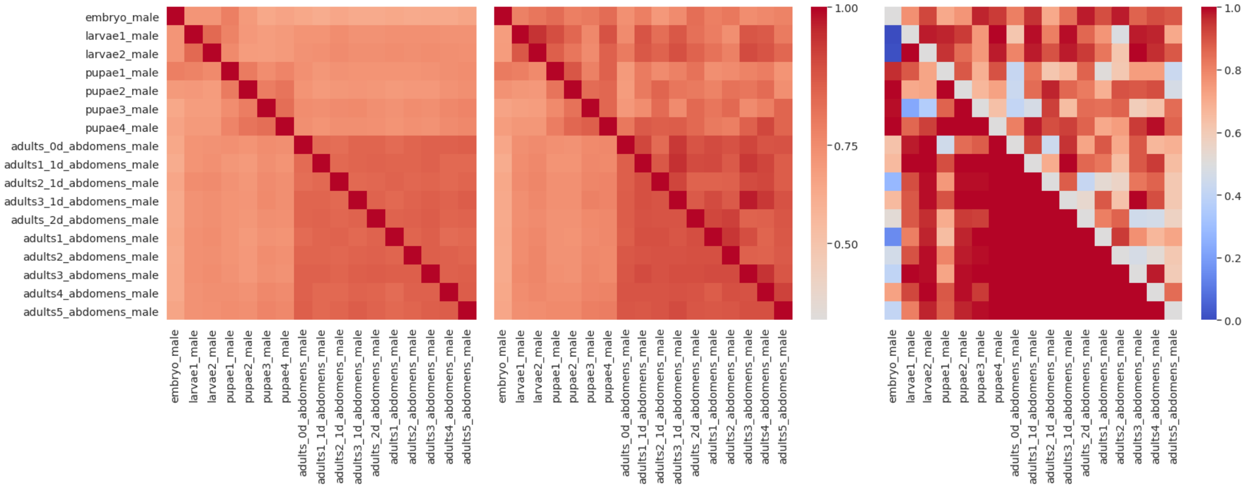

P. xylostella

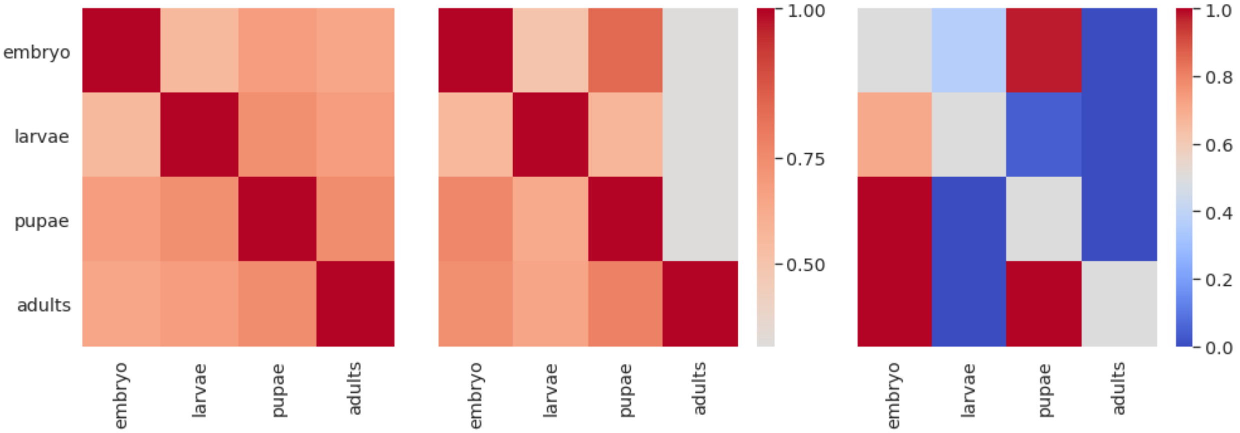

M. sexta, midgut samples

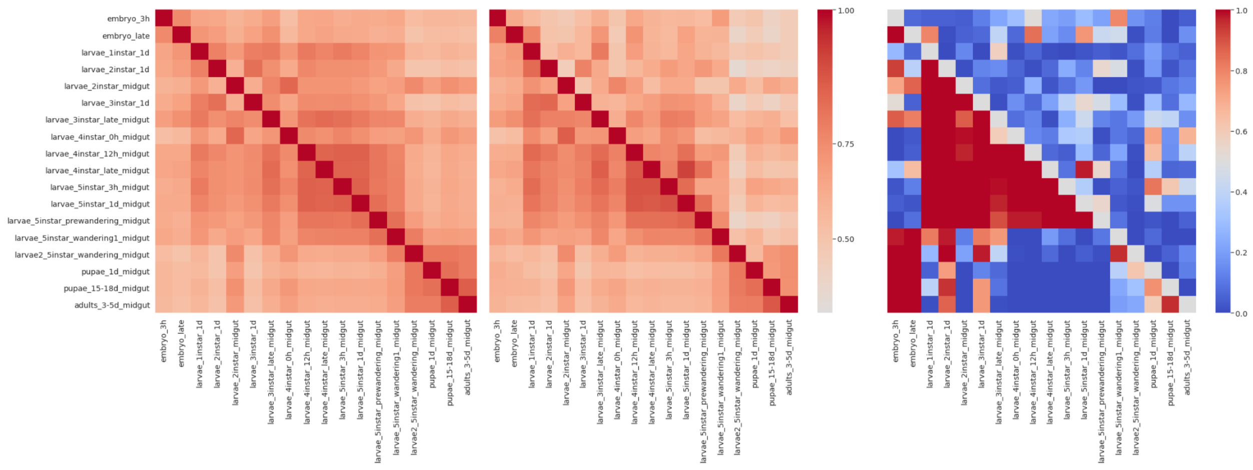

M. sexta, fat body samples

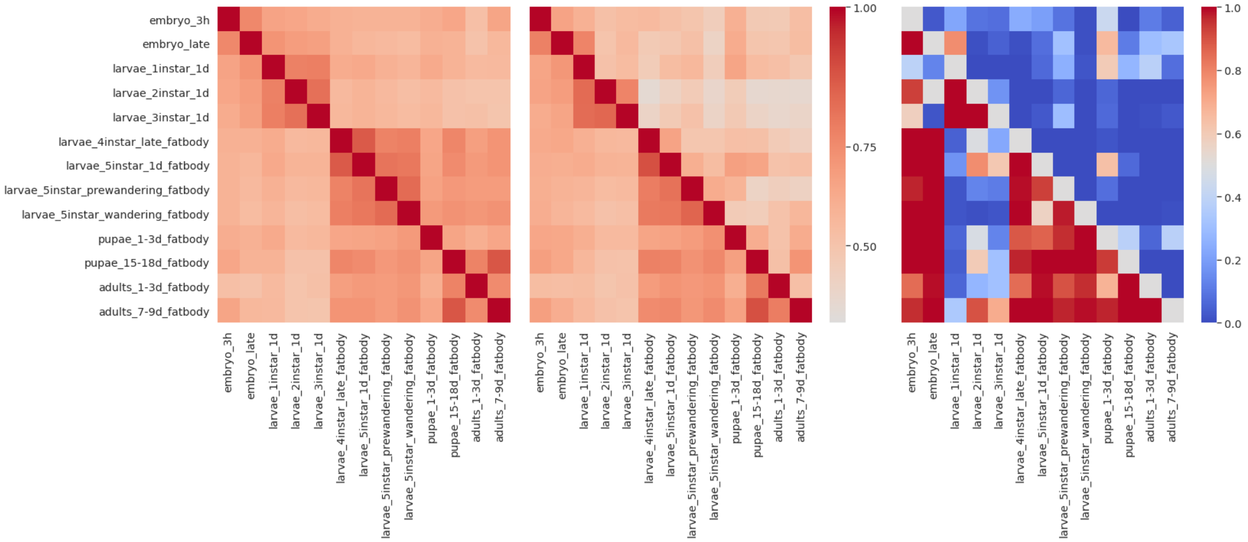

P. vanderplanki

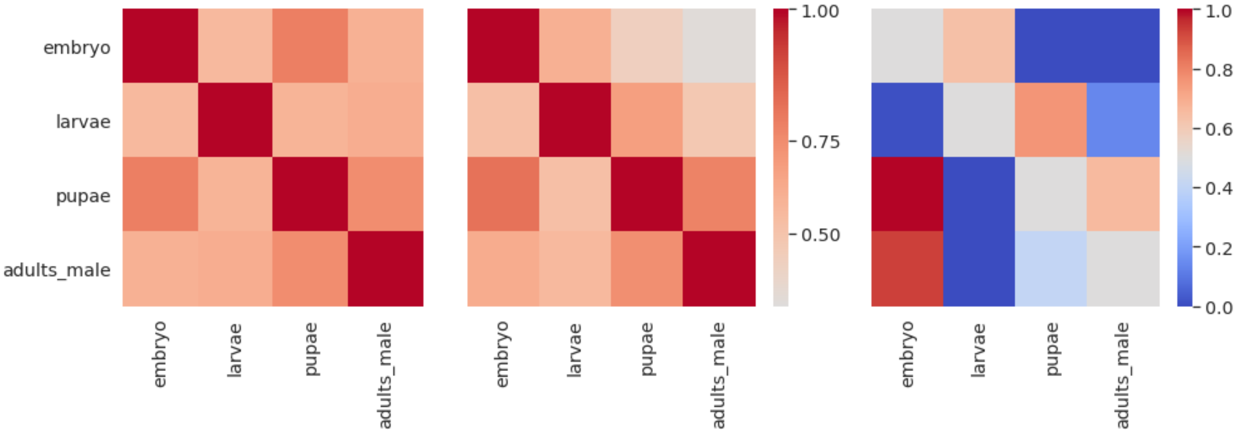

Supplement: Supplementary file 1 — Supplementary Information 1. [file 41598_2022_22188_MOESM1_ESM.pdf]
