# Supplementary material for: Recapitulation of the embryonic transcriptional program in holometabolous insect pupae
Source: Sci Rep. 2022 Oct 20;12:17570. doi: 10.1038/s41598-022-22188-y (PMC9584902; doi:10.1038/s41598-022-22188-y)

Supplementary Figure S2. Gene expression patterns across the development.

Genes from datasets with more than four measured time points were hierarchically clustered with the Spearman correlation coefficient as the distance metric. For each dataset, expression profiles for genes comprising 10 clusters are presented. Sequential stages of development are shown on the horizontal axis, log-transformed expression values — on the vertical axis. Median profile is shown in red.

D. melanogaster, Daines

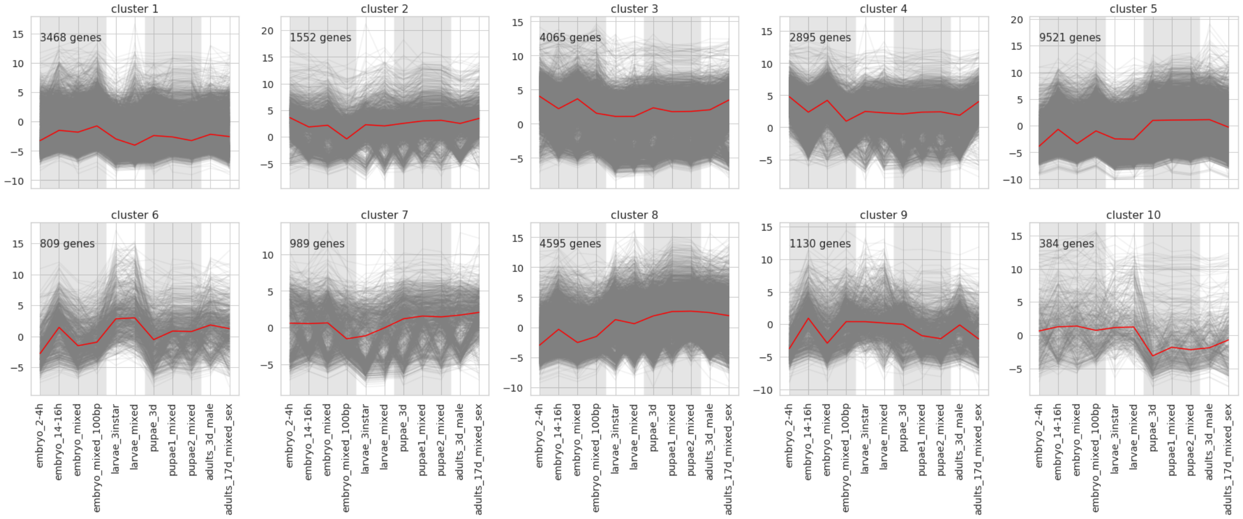

D. melanogaster, Graveley

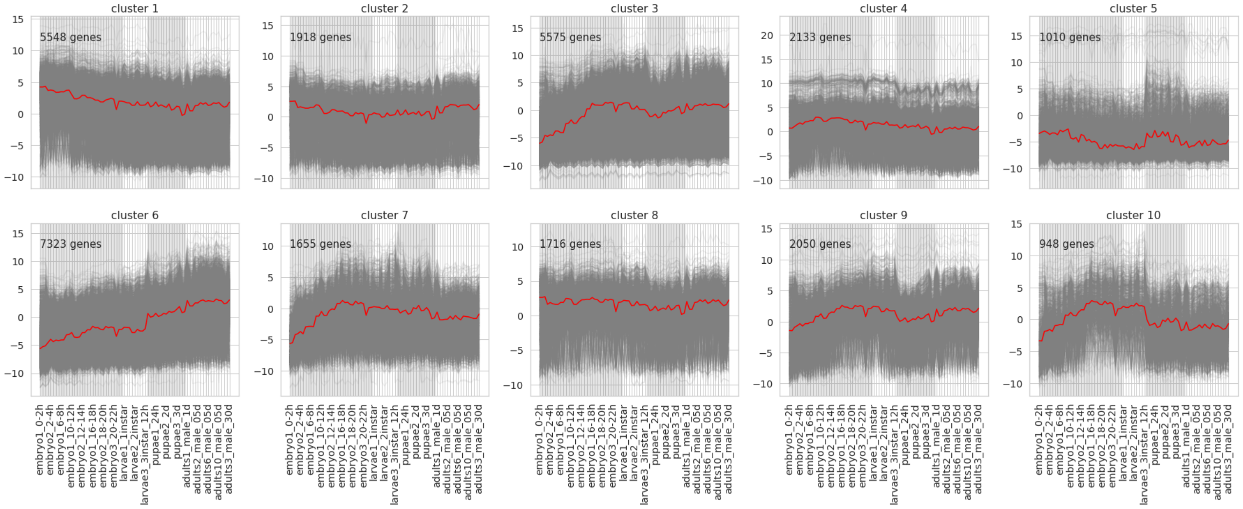

D. melanogaster, Arbeitman, GPL2837

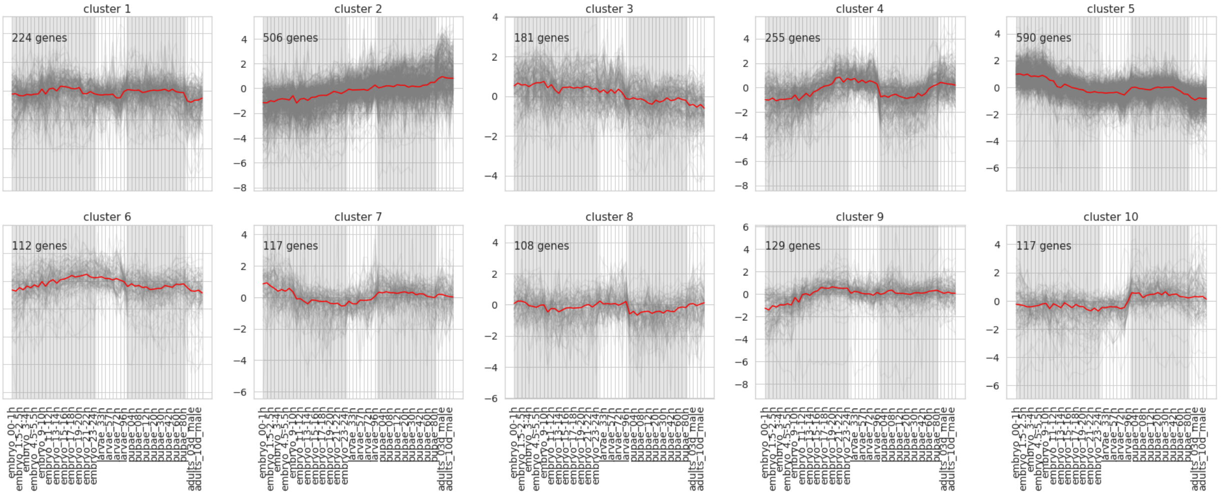

D. melanogaster, Arbeitman, GPL2838

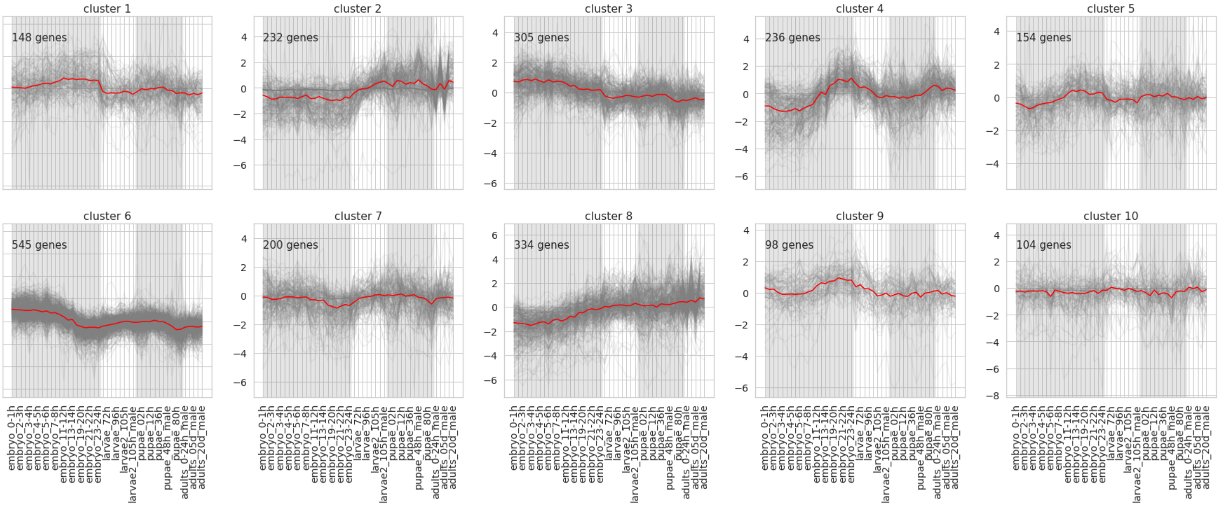

D. melanogaster, Arbeitman, GPL2840

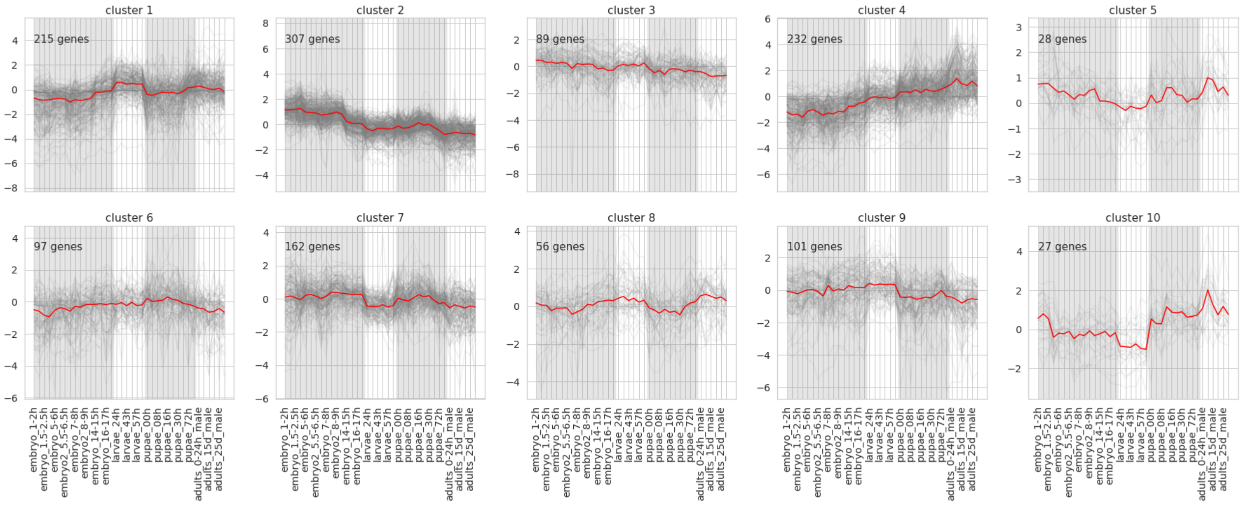

Z. cucurbitae, Wei

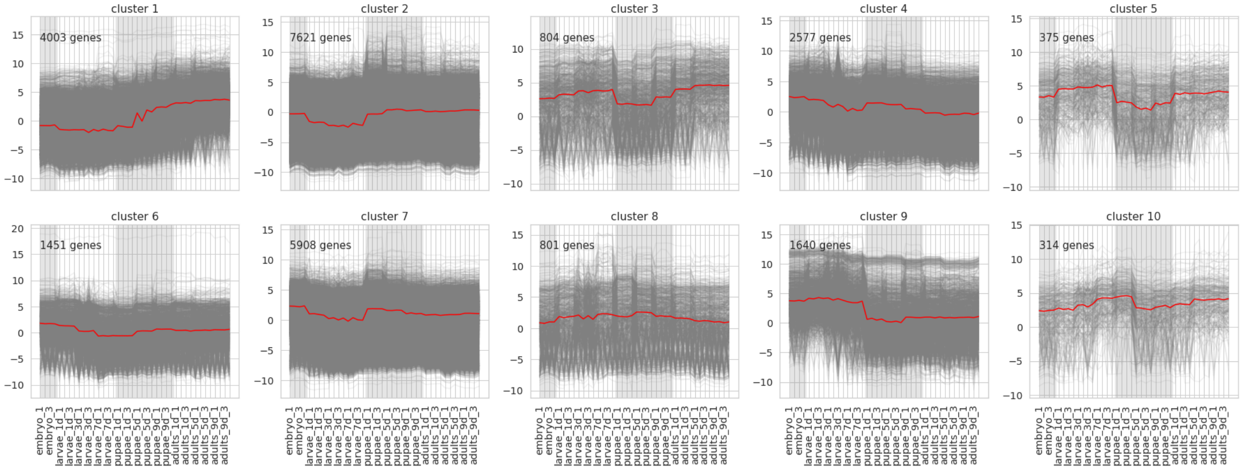

M. sexta, midgut samples

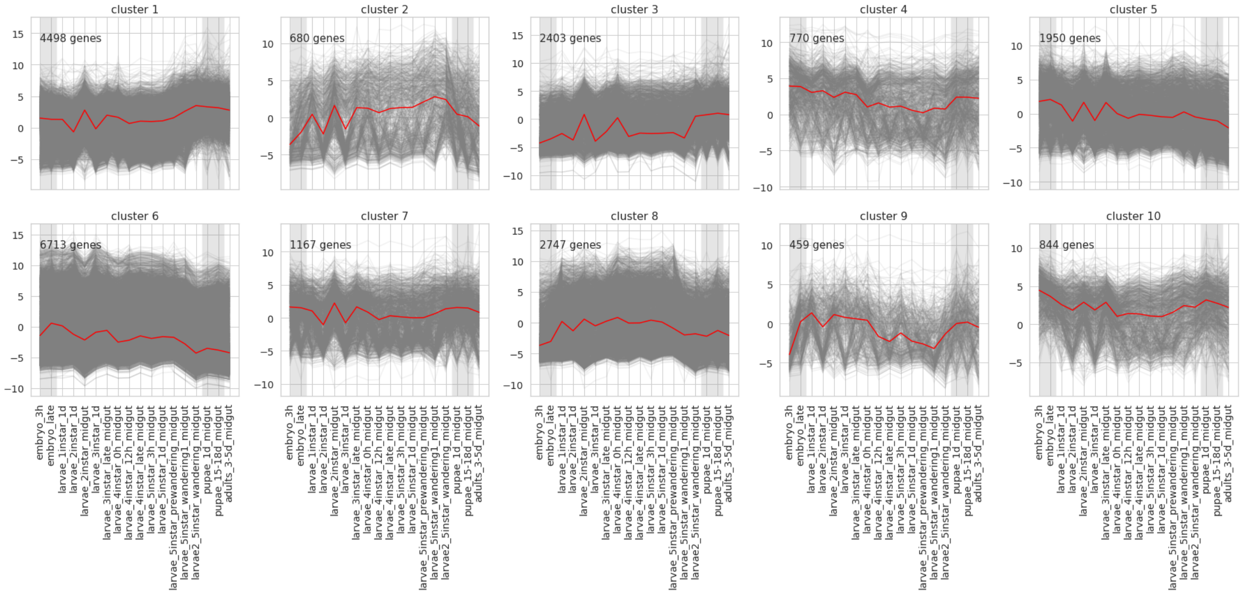

M. sexta, fat body samples

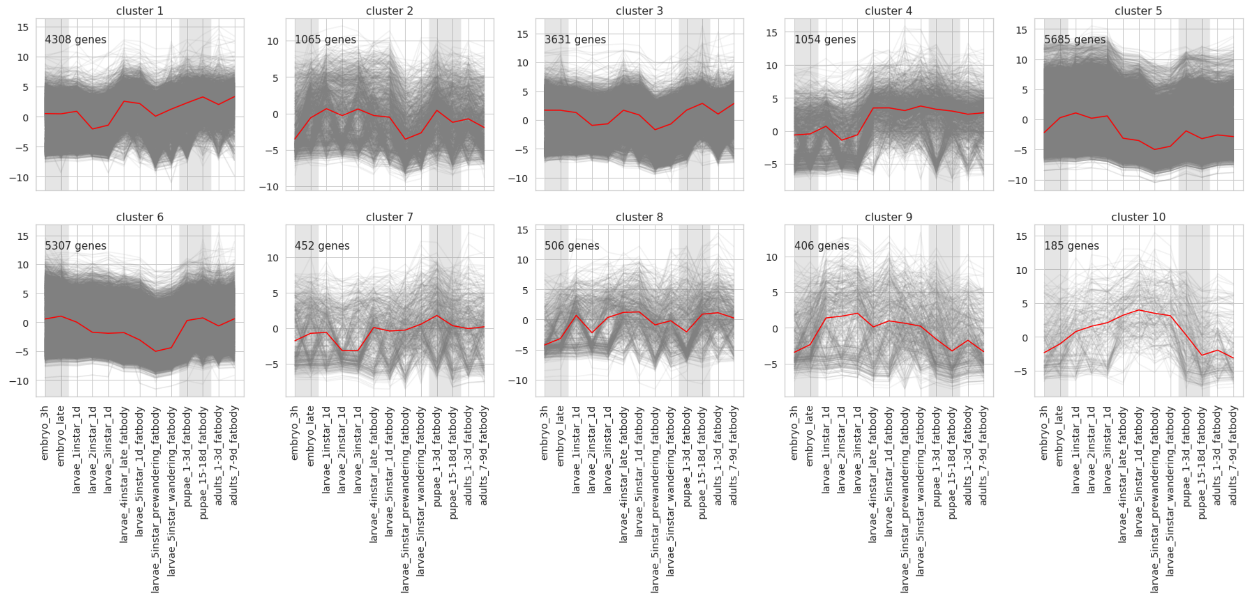

Supplement: Supplementary file 2 — Supplementary Information 2. [file 41598_2022_22188_MOESM2_ESM.pdf]
